# Gene-Edited Interleukin CAR-T Cells Therapy in the Treatment of Malignancies: Present and Future

**DOI:** 10.3389/fimmu.2021.718686

**Published:** 2021-07-27

**Authors:** Zhengchao Zhang, Lele Miao, Zhijian Ren, Futian Tang, Yumin Li

**Affiliations:** ^1^Department of General Surgery, Second Hospital of Lanzhou University, Lanzhou, China; ^2^Key Laboratory of Digestive System Tumors of Gansu Province, Second Hospital of Lanzhou University, Lanzhou, China

**Keywords:** CAR-T cells, interleukin, gene-edited, immunotherapy, TME, malignant tumor

## Abstract

In recent years, chimeric antigen receptor T cells (CAR-T cells) have been faced with the problems of weak proliferation and poor persistence in the treatment of some malignancies. Researchers have been trying to perfect the function of CAR-T by genetically modifying its structure. In addition to the participation of T cell receptor (TCR) and costimulatory signals, immune cytokines also exert a decisive role in the activation and proliferation of T cells. Therefore, genetic engineering strategies were used to generate cytokines to enhance tumor killing function of CAR-T cells. When CAR-T cells are in contact with target tumor tissue, the proliferation ability and persistence of T cells can be improved by structurally or inductively releasing immunoregulatory molecules to the tumor region. There are a large number of CAR-T cells studies on gene-edited cytokines, and the most common cytokines involved are interleukins (IL-7, IL-12, IL-15, IL-18, IL-21, IL-23). Methods for the construction of gene-edited interleukin CAR-T cells include co-expression of single interleukin, two interleukin, interleukin combined with other cytokines, interleukin receptors, interleukin subunits, and fusion inverted cytokine receptors (ICR). Preclinical and clinical trials have yielded positive results, and many more are under way. By reading a large number of literatures, we summarized the functional characteristics of some members of the interleukin family related to tumor immunotherapy, and described the research status of gene-edited interleukin CAR-T cells in the treatment of malignant tumors. The objective is to explore the optimized strategy of gene edited interleukin-CAR-T cell function.

## Introduction

CAR-T cells technology has achieved gratifying results in the clinical treatment of hematologic malignancies (1, 2). However, it has hit a bottleneck in treating solid tumors (3–6). Studies have shown that the inhibitory tumor microenvironment (TME) of solid tumors can inactivate CAR-T cells (7). The full activation and amplification of normal T cells require not only T cell receptor signals and costimulatory signals, but also the synergistic action of immune cytokines. Current theories suggest that the immunosuppressive TME of solid tumors is mainly characterized by the suppression of immune cell function, So it weakens CAR-T cells tumor immunity (8, 9). To overcome this challenge, multiple strategies have been applied to optimize CAR-T cells technology. Immune cytokines are the basis of T cells’ immune function, and they have been demonstrated that they can significantly improve the antitumor activity of CAR T cells (10). Therefore, the researchers created a fourth generation of CAR-T cells by gene modifying the structure of CAR-T cells using immune cytokines (11, 12).

Interleukin is a type of cytokine produced by multiple immune cells and used by these immune cells. Some members of the interleukin family exert multifarious roles in the anti-tumor process as growth factors of T cells. At present, many gene-edited interleukin CAR-T cells have achieved positive efficacy in the treatment of malignant tumors in preclinical studies, and related clinical studies are ongoing. With the structural optimization of gene-edited interleukin CAR-T cells, its efficacy in overcoming the immunosuppressive TME is also increasing. Here, we shown the correlation between the above families of interleukin and tumor immunotherapy, and summarize the research progress of their application for CAR-T cells technology. Finally, the optimization of gene-edited interleukin-CAR T cells in anti-tumor therapy was discussed.

## Members of The Interleukin Family and Tumor Immunity

Last decade, with the development of tumor immunotherapy, the function of interleukin in tumor immunotherapy has attracted more and more attention from researchers. A large number of tumor immunotherapy techniques began to use interleukin to improve the immune response of tumor. **Table 1** shows part of the interleukin family and their functions related to tumor immunotherapy.

**Table 1:** Summary of cytokines related to tumor immunotherapy in the interleukin family.

Interleukins	Tumor immune-related functions	Receptors	The associated immune cells	Associated activation pathway
IL-1 family				
IL-1	Proinflammatory, regulating adaptive immune response	IL-1R	DCs, T cells	NF-κB (13)
IL-18	T cell are activated by enhancing endogenous TCR	IL-18Rα/IL-18Rβ	CD8 ^+^T cells, NK cells	NF-κB (14)
IL-33	Bidirectional regulation of tumor immune response	ST2	Th cells, NK cells, Treg cells	NF-κB,MAP (15)
IL-36	Promote DCs maturation and indirectly promote T cell proliferation	IL-36R	DCs,T cells	NF-κB,MAP (16)
IL-2 family				
IL-2	Regulate the proliferation and apoptosis of activated T cells	IL-2Rα/IL-2Rβ	T cells, NK cells, monocyte macrophages, B cells	STAT5 (17–19)
IL-4	Regulates the function of Th1 and Th2 cells	IL-4R	Th cells,	STAT6 (20)
IL-7	Promote T cell proliferation and maintain cell homeostasis	IL-7Rα	Naive and memory T cells	STAT5 (21, 22)
IL-9	Promote the proliferation and activation of T cells	IL-9R	CD8+ T cells, NK T cells	STAT1, STAT3, STAT5 (23)
IL-15	Promote T cell proliferation and maintain cell homeostasis	IL-15Rα/IL-2Rβ	CD8 +T cells,NK cells	STAT5 (24)
IL-21	Modulate effector function of CD8+ T cells and polarization of CD4+ T Th cells	IL-21R	CD8+ T cells, CD4+ T cells, NK T cells	STAT3 (25, 26)
IL-6/12 family				
IL-6	Regulates immune response and inflammation	IL-6R	T cells	STAT3 (27)
IL-12	Enhance the IFN-γ secretion function of Th17 cells and cytotoxic effect of NK cells and T cells, stimulate T cell differentiation	IL-12Rβ1/IL-12Rβ2	NK cells, NK T cells, CD8+T cells	STAT4 (28)
IL-23	Promotes memory T cell proliferation	IL-23R	T cells	STAT3 (29)
IL-27	Affects antigen presentation and regulates the differentiation and activation of Th cells	gp130/WSX-1	Treg cells	STAT1, STAT3 (30)
IL-35	Promotes immunosuppression by inhibiting the differentiation of Th1 and Th17 cells	IL-12Rβ2/gp130/WSX-1	Treg cells	STAT1, STAT3, STAT5 (30)

### Correlation Between IL-1 Family Members and Tumor Immunity

The IL-1 family mainly includes IL-1, IL-18, IL-33, and IL-36. They initiate a powerful inflammatory and immune response by binding to specific receptors in the IL-1 receptor family. These immunomodulatory molecules are generated by immune cells and regulate the function of these immune cells. Therefore, they are closely related to tumor immunity.

IL-1 is a pro-inflammatory cytokine, which includes two subtypes of IL-1α and IL-1β, and regulates adaptive immune response mainly through binding with its receptor (IL-1R) in the body. IL-1α acts as a local alarm in the event of cell damage, while IL-1β release can also occur in the circulation and is strictly controlled. IL-1β is primarily derived from myeloid cells and is upregulated and associated with disease progression in many different types of cancer, such as colon and lung malignancies. Cancer cells also drive tumor-associated inflammatory macrophages to produce IL-1β, which inhibits tumor immune response through IL-1β-mediated accumulation of myeloid derived suppressor cells (MDSCs). Therefore, current clinical studies have focused on the role of antagonistic IL-1 β activity in anti-tumor (13). These results indicate that IL-1β acts on adaptive immunity and may indirectly modulate T cell immune response to tumor.

IL-18 is also an important pro-inflammatory and immunomodulatory cytokine (31), which activates T cell proliferation and IFN-γ secretion by enhancing endogenous TCR. It can also promote more effective tumor killing by enhancing the expression of Fas ligands in immune cells (32). Besides, studies have demonstrated that IL-18 improves T cell function without causing severe dose-limiting toxicity (33, 34). Therefore, IL-18 is a promising candidate cytokine for gene-edited CAR-T cells.

As an inflammatory factor, IL-33 plays multiple roles in tumor immunity. In 2015, a study found that IL-33 was identified as a ligand for oncogenic inhibitory receptor 2 (ST2) (35). IL-33 plays an immunomodulatory role by interacting with ST2. IL-33 can act on multitudinous immune cells, such as Th1, Th2, NK and regulatory T cells (Tregs) (15). Therefore, IL-33 has a bidirectional regulatory function of different cancer immune cells. Three subtypes of IL-36, known as IL-36α, IL-36β, and IL-36γ, have different functions. IL-36 has been shown to promote upregulation of CD80 and CD86, markers of DCs activation, and promote DCs maturation (36). The immunoregulatory function of IL-36α is to directly promote the proliferation of CD4+T cells (37). IL-36β promotes T cell proliferation by promoting the production of IL-12 and IL-18 by DCs (38). The function of IL-36γ is to induce CD4+T cells to secrete IFN-γ, IL-4 and IL-17 (39).Therefore, IL-36 also exerts a bidirectional regulatory role in the process of tumor immunity, and has both activation and inhibition effects.

### Correlation Between IL-2 Family Members and Tumor Immunity

The IL-2 family is part of the receptor γc family, which belongs to type I cytokines, and they contain many interleukins. Its members mainly include IL-2, IL-4, IL-7, IL-9, IL-15, and IL-21,and all of them play immunomodulatory functions through the JAK-STAT pathway (40, 41). And these cytokines exert vital functions in the regulation of immune cells.

IL-2 is a T cells growth factor that enhances the cytolytic activity of NK cells (17). It promotes Tregs differentiation, which regulates the adaptive immune response (18). At present, IL-2 is the main cytokine used to culture T cells for immunotherapy. Nevertheless, T cells cultured by IL-2 showed phenotypic heterogeneity and were mainly composed of effector memory cells that had full functional effects but were sensitive to death. IL-4 is mainly involved in the function regulation of Th2 cells, so it is known as Th2 cytokine. It can promote tumor progression by down-regulating Th1 signaling and directly inactivating CD8+T cells (42). Shuku-ei Ito et al. investigated the effect of neutralizing IL-4 on tumor immunity (20), the results suggested that an IL-4 antibody can enhance anti-tumor immunity. Therefore, IL-4 can be used as a target for tumor immunotherapy due to its role in the tumor microenvironment.

IL-7 is the most important tumor immune-related cytokine in the γc family, and its function is mainly to regulate naive T cells and memory T cells homeostasis (21, 22). Studies have confirmed that IL-7-induced signal transduction defect is the main reason for affecting T cell development in severe combined immunodeficiency disorder (SCID) patients (43) and in patients with SCID caused by JAK3 mutation (44, 45). IL-7 is an indispensable cytokine for T cell growth, therefore, IL-7 has also become a popular cytokine in gene-edited CAR-T cells research. IL-9 is also an important tumor immune-related cytokine, mainly produced by Th9 cells (46, 47). IL-9 derived from Th9 cells can improve the tumor killing function of CD8+ T cells and NK T cells by promoting secretion of IFN-γ (48, 49). Therefore, Th9 cells have been shown to have an antitumor effect in most solid tumors (50). However, it has been shown to be tumorigenic in most hematologic tumors (51).

As an immunoregulatory cytokine, IL-15 is an important homeostasis cytokine of CD8+T cells and NK cells. The main function of IL-15 is to promote the growth of memory CD8+T cells (52, 53). Therefore, L-15 has been used in several studies to optimize the structure of CAR-T cells. However, IL-15 must form the IL-15/IL-15Rα complex in order to exert its tumor immune function. IL-15/IL-15Rα complex has poor stability and can bind to IL-15Rβγ to decrease tumor immune efficacy (54). Therefore, the stability of IL-15/IL-15Rα complex is essential for IL-15 to perform tumor immune function. The researchers used several strategies to improve the stability of IL-15 function. One strategy is to extend the persistence of the IL-15/IL-15Rα complex by fusion with the IgG Fc domain, resulting in more persistent induction of CD8+T cells and NK cells (55). Another strategy is to enhance the capability of IL-15 through a fusion protein that is conjugated to human IL-15 through the ligosome in the terminal cytokine binding domain of human IL-15RαNH_2_ and has similar biological activity to that described above (54).

IL-21 is a multifunctional cytokine, exerts a vital role in regulating the function of CD8+ T cells (25). IL-21 can improve the activity of CD8+ T cells, making it potentially valuable in cancer immunotherapy (56). Besides, a recent study on pancreatic cancer found that IL-21 also has an anti-tumor effect by enhancing NK cell function (57). IL-21 has also been used in studies of CAR-T for its ability to positively regulate tumor-associated immune cells.

### Correlation Between IL-6/12 Family Members and Tumor Immunity

The family members include typical members IL-6, IL-12, IL-23, IL-27, and IL-35. Cytokines in the IL-12 family influence the outcome of cancer, infection, and inflammatory disease. Most of the members are produced by DCs, macrophages, endothelial cells, T lymphocytes, and tumor cells (58),which conduct downstream signal transduction through JAK protein and STAT. They regulate tumor immunity in both direct and indirect ways.

IL-6 is a pleiotropic cytokine,affects T cell activation, amplification, survival, and polarization (59). Studies have shown that during the inflammatory process, IL-6 signaling has been found to promote the expression of T cell attractor chemokines (60). IL-6 can also regulate the surface expression of Fas receptor through up-regulating anti-apoptotic factors by STAT3, thereby inhibiting T cell apoptosis (61, 62). IL-6 has been also demonstrated to participate in the accumulation of MDSCs in tumors (29). In addition, IL-6 exerts vital roles in the acute immune response. When stimulated by local inflammation, IL-6 can promotes the production of acute phase proteins by acting on the liver (63). IL-6 is an important factor affecting liver cells, hematopoietic progenitor cells, cardiovascular, endocrine and nervous system homeostasis (64). Therefore, a large number of CAR T clinical trials have shown that high serum IL-6 levels are associated with cytokine release syndrome (CRS), and IL-6 is a monitoring indicator in the clinical diagnosis and treatment of CRS (65).

As an inflammatory cytokine, IL-12 is mainly generated by DCs cells and macrophages. Studies have demonstrated that IL-12 can improve the activation of Th1 and Th17 cells (66) and enhance the cytolysis ability of CD8+T cells (67). Therefore, IL-12 is expected to be successful in adoptive immunotherapy of tumors due to its positive regulation of tumor immune properties. IL-23 is constituted of IL-23αp19 and IL-12βp40 (29), and facilitates the proliferation of memory T cells, especially Th17 cells expressing the its receptor (IL-23R) (68–70). IL-23 activates the tumor immune response to inhibit tumor progress, which has given rise to the application of IL-23 in the treatment of tumors by gene-edited CAR-T.

IL-27 is an effective immunomodulatory cytokine, which mainly has anti-inflammatory and inhibitory properties in immunomodulatory regulation, especially in inhibiting Th2 and Th17 differentiation. However, recent studies comparing these results have also demonstrated that IL-27 promotes the growth and survival of Tregs (30). Myeloid and epithelial cells treated with IL-27 also showed enhanced antigen presentation by upregulating MHCI and MHCII as well as costimulatory molecules (71). Therefore, IL-27 is also a major regulator of TME. IL-35 is an effective regulatory cytokine, mainly secreted by Tregs. IL-35 can convert T cell into the regulatory cell population that produces IL-35, which is called the induction of Tregs-IL-35 (69, 72). IL-35 inhibited function of Th1 and Th17 cells by promoting the expansion of Tregs (72, 73). Therefore, IL-35 is an immunosuppressive cytokine and exerts important roles in promoting tumor progression.

## Correlation Study of Gene-Edited Interleukin CAR-T Cells In The Treatment Of Malignant Tumors

The researchers genetically engineered these cytokines to modulate CAR-T activity to better kill tumor cells. At present, a great number of preclinical studies have confirmed that gene-edited co-expression of cytokines such as IL 7, IL 12, IL 15, IL 18, IL21, and IL 23 can enhance the antitumor activity of CAR-T (**Table 2**). Simultaneously, clinical trials of gene-edited interleukin-CAR-T for malignancies are under way at several medical centers around the world (**Table 3**), involving hematological tumors and solid tumors, to evaluate its effective dose and safety.

**Table 2:** Summary of preclinical studies on the use of CAR-T cells co-expressing cytokines in the treatment of malignant tumors.

Tumor	Targeted antigen	Gene-edited cytokines	Reference
Lung cancer, pancreatic ductal adenocarcinoma	hCD20, Mesothelin	IL-7 and CCL19	Keishi Adachi et al. (74)
prostatic cancer	NKG2D	IL-7	Cong He et al. (75)
hepatic carcinoma	GPC3	IL-7 and PH20	Xingcheng Xiong et al. (76)
breast carcinoma	AXL	C7R	Zhenhui Zhao et al. (77)
Colorectal cancer, pancreatic cancer, stomach cancer	CEA	IL‐12	Xiaowei Chi et al. (78)
lymphoma	CD19	IL-12	Gray Kueberuwa et al. (79)
hepatic carcinoma	glypican-3 (GPC3)	IL-12	Ying Liu et al. (80)
ovarian cancer	Muc-16	IL-12	Oladapo O.Yeku et al. (81)
leukemia	CD19	IL-15	Lenka V. Hurton et al. (82)
Cerebral endothelioma	VEGFR-2	IL-15	Evripidis Lanitis et al. (83)
melanoma	CD19	IL-18	Biliang Hu et al. (84)
hepatic carcinoma	GPC3	IL-21	Yi Wang et al. (85)
chronic lymphocytic leukemia	CD19	IL-21	Štach M et al. (86)
hepatic carcinoma	GPC3	IL-15 and IL-21	Batra S. A et al. (87)
neuroblastoma	GD2	IL-23	Xingcong Ma et al. (88)
prostatic cancer	PSMA	IL-23	Dawei Wang et al. (89)
hepatic carcinoma	GPC3	4/21 ICR	Yi Wang et al. (85)
pancreatic cancer	PSCA	4/7 ICR	Somala Mohammed et al. (90)

**Table 3:** Clinical trial summary of gene-edited interleukin CAR-T cells.

Targeted antigen	Tumor	Gene-edited cytokines	Patients(n)	Clinical stage	Identifying code(ClinicalTrials.gov)	Sponsor	Status
EGFR	metastatic colorectal cancer	IL-12	20	I	NCT03542799	Shenzhen Second People’s Hospital, China	Not yet recruiting
CD19	Diffuse large B cell lymphoma	IL7 and CCL19	24	I	NCT04381741	The Second Affiliated Hospital of Zhejiang University, China	Recruiting
Nectin4/FAP	Nectin4 positive late malignant solid tumor	IL7 and CCL19, or IL12	30	I	NCT03932565	The Sixth Affiliated Hospital of Wenzhou Medical University, China	Recruiting
CD19	lymphoma	IL-7 and IL -15	20	I/II	NCT02652910	Xinqiao Hospital, Chongqing City, China	Unknown status
GD2	neuroblastoma	IL -15	18	I	NCT03721068	Rineberg Comprehensive Cancer Center, USA	Recruiting
CD19/CD20	lymphoma	IL-7 and IL-15	32	I/II	NCT04186520	Medical College of Wisconsin, USA	Recruiting
GD2	Neuroblastoma, osteosarcoma	C7R	94	I	NCT03635632	Baylor College of Medicine, USA	Recruiting
GD2	neuroglioma	C7R	34	I	NCT04099797	Baylor College of Medicine, USA	Recruiting
GPC3	Multiple solid tumors (liver cancer, sarcoma, etc.)	IL -15	24	I	NCT04377932	Baylor College of Medicine, USA	Not yet recruiting
GPC3	Multiple solid tumors (liver cancer, sarcoma, etc.)	IL -15 and IL-21	24	I	NCT04715191	Baylor College of Medicine, USA	Not yet recruiting
CD138, integrin β7, CS1, CD38 and BCMA	multiple myeloma	IL7 and CCL19	30	I	NCT03778346	The Sixth Affiliated Hospital of Wenzhou Medical University, China	Recruiting
CD19	lymphoma	IL -18	30	I	NCT04684563	University of Pennsylvania, USA	Not yet recruiting
CD5	T-cell Acute Lymphoblastic Leukemia T-cell Non-Hodgkin Lymphoma	IL15/IL15 sushi	20	I	NCT04594135	Peking University Shenzhen Hospital Shenzhen, Guangdong, China	Recruiting
MUC16	Multiple solid tumors	IL-12	18	I	NCT02498912	Kettering Cancer Center, USA	Active, not recruiting

All clinical trials were download at www.clinicaltrials.gov (access date: March 04, 2021).

### IL-7

IL-7 has been widely used in tumor immunotherapy to enhance the anti-tumor immune response of T cells (91, 92). Studies have shown that IL7 not only promotes CD8+ T cell proliferation and reduces T cell apoptosis and depletion by enhancing Bcl-2 expression, but also increases the phenotype of poorly differentiated CAR-T cells, thus improving the persistence and viability of CAR-T cells (75, 93). There were also clinical trials (NCT00586391, NCT00709033) that amplified CAR-T cells with IL-7 and IL15 *in vitro*, and then confirmed these findings by phenotypic analysis of CAR-T cells (94).Cong He et al. (75)constructed gene-edited IL-7 CAR-T cells targeting NKG2D, and found that co-expressing IL-7 enhanced the proliferation and persistence of NKG2D-CAR-T cells *in vitro* and *in vivo*. In order to further optimize the construction of CAR-T cells, researchers have used IL-7 in combination with other cytokines to modify CAR-T cells, and achieved promising results in preclinical study. For instance, Keishi Adachi et al. (74) constructed CAR-T cells that co-expressing IL-7 and CCL19, and found that multiple cytokines significantly improved tumor infiltration and survival of CAR-T cells. More robust antitumor activity and durability than conventional CAR-T has been realized in studies targeting solid malignant tumors. These related clinical trials are ongoing, such as targeting CD19 CAR-T trial for lymphoma (NCT04381741); targeting NECTIN4/FAP CAR-T for advanced malignant solid tumors (NCT03932565). Similarly,Xingcheng Xiong et al. (76) constructed CAR-T cells co-expressing IL-7 and hyaluronidase(PH20) in the preclinical study of targeting GPC3 CAR-T cells for liver cancer, and the results showed that the co-expression of IL-7 and PH20 may obviously improve the efficacy of CAR-T cells for solid tumors. Other clinical studies of co-expressing IL-7 and IL-15 CAR-T cells for lymphoma are also ongoing (NCT02652910, NCT04186520), aiming to test the hypothesis that co-expressing IL-7 and IL-15 CAR-T cells persist for longer after infusion in patients with lymphoma. And whether the persistence of CAR-T cells improves the anti-lymphoma efficacy.

Furthermore, IL-7 receptor (C7R) was also used for the construction of gene-edited CAR-T. A recent study confirmed the significant antitumor activity of co-expressing C7R CAR-T cells against neuroblastoma and glioblastoma (95). Two clinical trials (NCT03635632, NCT04099797) of CAR-T co-expressing C7R targeting GD2 in the treatment of neuroblastoma, osteosarcoma, and glioma are currently under way, the purpose of the studies was to find the maximum safe dose of GD2-C7R CAR-T cells and assess how long they can be detected in the blood and their effect on tumors.

### IL-12

Because IL-12 can effectively mobilize the immune system, it has become one of the cytokines that mediate anti-tumor activity (96–98). A series of preclinical studies have demonstrated that IL-12 has antitumor activity by degrading tumors or prolonging survival in tumor-bearing animals (99). Giulia Agliardi et al. (100) conducted a preclinical study on the treatment of glioblastoma multiforme (GBM) by combining CAR-T cells with local injection of IL-12. The results showed that CAR-T therapy combined with local injection of IL-12 resulted in a more durable antitumor response than CAR-T therapy alone. The study also demonstrated that IL-12 not only enhanced the cytotoxicity of CAR-T cells, but also remodeled TME, promoted the infiltration of pro-inflammatory CD4+ T cells, and reduced the number of Tregs. However, systemic use of IL-12 can cause serious and unexpected side effects, which greatly limits its clinical use (101, 102). In the face of this challenge, the researchers have been trying to construct gene-edited IL-12 CAR-T cells in an effort to enhance anti-tumor activity while mitigating its side effects (103, 104). Ying Liu et al. (80) designed targeting GPC3 CAR-T cells and IL12-GPC3-CAR-T cells. This study demonstrated that IL12-GPC3-CAR-T cells were more capable of lysis of GPC3+ tumor cells and secreted more cytokines than GPC3-CAR-T cells. IL-12-GPC3- CAR-T cells showed a stronger antitumor effect in tumor-bearing mice due to increased infiltration and persistence of T cells by IL-12. Similarly, Gray Kueberuwa et al. (79) used CAR-T cells expressing murine IL-12 (IL12-CD19-CAR-T cells) to show eradication of B-cell lymphoma with a long-term survival rate. They also demonstrated that IL12-CD19-CAR-T cells not only kill CD19+ tumor cells directly, but also recruit host immune cells for an anticancer immune response. This finding may enable gene-edited IL-12 CAR-T cells to be used in the treatment of malignancy without the need for lymphatic clearance, so that these cells can be better used for anti-tumor immunity.

Fengtao You et al. (105) constructed CAR T cells targeting MUC1 co-expressing IL-12 (MUC1-IL-12-CAR T cells) and targeted CAR T cells modified with MUC1 (MUC1-CAR T cells) for use in seminal vesicle carcinoma in Phase I clinical trials (NCT02587689). MUC1-IL-12-CAR-T cells using MUC1 normal SCFV sequence SM3; MUC1-CAR T cells use the mutated SM3 scFv sequence. Two CAR T cells were injected locally into two separate metastatic lesions of the same seminal vesicle carcinoma patient. The results showed that MUC1-CAR T cells effectively induced tumor necrosis, while MUC1-IL-12 CAR T cells treated lesions showed no tumor necrosis. Of course, the purpose of this clinical study was to demonstrate the importance of SCFV in CAR T cell therapy. But it also demonstrated the safety of gene-edited IL-12 CAR T cells for clinical use. Two clinical trials (NCT03542799 and NCT02498912) are currently evaluating the safety and feasibility of co-expressing IL-12 CAR-T cells in patients with solid tumors, as well as evaluating the maximum tolerated dose.

### IL-15

The tumor immune function of IL-15 is mainly to maintain CD8+ memory T cell homeostasis and inhibit activation-induced cell death (106). Therefore, gene-edited IL-15 CAR-T cells have been demonstrated to be superior in the treatment of malignant tumors. Evripidis Lanitis et al. (83) used retroviral vectors to encode co-expressed mouse interleuk-15 CAR-T cells (IL-15-CAR-T) targeting tumor blood vessels. Results showed that co-expression of IL-15 not only enhanced the tumor infiltration and control of tumor growth, but also enhanced the effect of IL-15 on tumor growth. Furthermore, TME was optimized (activation of NK cells and reduction of M2 macrophages). Further studies showed that the expression of Bcl-2 in CAR-T cells expressing IL-15 was up-regulated, while the expression of PD-1 was down-regulated. Analogously, Lenka V. Hurton et al. (82)designed co-expressing IL-15 CAR-T cells using gene-edited technology, which demonstrated a strong killing effect against CD19+ leukemia in preclinical experiments. The study analyzed the phenotype of proliferating T cells and found that the most persistent T cell phenotype was consistent with that of T memory stem cells. The results demonstrated that IL15 signaling could maintain T memory stem cells persistence. Which lays a theoretical foundation for the further application of IL-15 in optimizing CAR-T cells construction.

Gene-edited IL-15 has also shown enhanced antitumor activity of CAR T cells in clinical trials. Jia Feng et al. (107) modified CD5-targeted CAR-T cells by means of genetic engineering to secrete IL-15/IL-15 Sushi(IL-15 protein linked to the IL-15Rα sushi domain of the IL-15 receptor) Complex. In a phase I clinical trial (NCT04594135), these CAR-T cells were tested for safety and efficacy in a patient with refractory lymphoblastic lymphoma with central nervous system infiltration. In the trial, symptoms of central nervous system compression were significantly reduced after 3 weeks of treatment with IL-15-CD5-CAR-T cells, and soft tissue mass shadow was significantly reduced after 8 weeks of treatment. These results suggest that gene-engineered IL-15 CAR-T cells are an effective treatment for T cell malignancies, especially in patients with central nervous system involvement. At present, clinical trials (NCT03721068, NCT04377932) are under way to treat multiple solid tumors (liver cancer, sarcoma, fibroblastoma). The goal of these studies is to determine the maximum safe dose of CAR-T cells and how long they last in the body. To understand the side effects and evaluate its efficacy in solid tumors.

### IL-18

Previous studies have shown that the structural expression of IL-18 by CAR-T cells significantly enhances the antitumor activity of CAR-T cells (84). Biliang Hu et al. (84) constructed CD19-IL-18 CAR-T cells using transgenic technology to conduct *in vivo* anti-tumor studies. CD19-IL-18 CAR-T cells significantly enhanced the proliferation of CAR-T cells. And effectively enhance the anti-tumor effect of melanoma mice. The study confirmed that the proliferation of IL-18-secreting CAR- T cells in the transplanted model was significantly enhanced, which was dependent on the IL-18R signaling pathways. This finding provides a strategy for the use of CAR-T cells in solid tumors. Since, Yong Huang et al. (14) also found that exogenous IL-18 could improve the anti-tumor function of HER2-specific CAR-T cells *in vitro* and *in vivo*, not only in immunodeficient mice, but also in immunotolerant mice. In addition, Markus Chmielewski et al. (108) found that the anti-tumor process of CAR-T cells co-expressing IL-18 was accompanied by the overall change of tumor immune microenvironment. Specifically, the number of M1 macrophages and NK cells increased, while the number of Tregs, inhibitory DC and M2 macrophages decreased, indicating that IL-18 has the function of recruiting peripheral immune cells to participate in anti-tumor combat. University of Pennsylvania team is currently conducting a clinical trial (NCT04684563) of co-expressing IL-18 CAR-T cells targeting CD-19 in the treatment of lymphoma. The primary objective of this study is to evaluate the maximum safe dose.

### IL-21

IL-21 can enhance tumor immune response mediated by T cells. Li Du et al. (109) found that the addition of IL-21 in the preparation of CAR-T cells could improve the T cell transfection efficiency by reducing the expression of IFN-γ in activated T cells. They also shown that exogenous IL-21 improved the cytotoxicity of CAR-T cells by enhancing the enrichment and amplification of poorly differentiated CAR-T cells. This finding lays a foundation for the application of IL-21 to optimize the structure of CAR-T cells. ŠTach, M et al. (86) constructed gene-edited IL-21 CAR-T cells targeting CD19, and studied the effect of IL-21 on its function. The results showed that IL-21 enhanced the expansion of CAR-T cells, and prevented the differentiation of CAR-T cells into late memory phenotype. Besides, gene-edited IL-21 promoted tumor infiltrating of CD19 CAR-T cells, leading to tumor growth retarded. Yi Wang et al. (85) constructed 4/21 ICR-CAR-T cells and reversed the efficacy of IL-4 against CAR-T cells in the environment of hepatocellular carcinoma(HCC) through the IL-21 pathway. The 4/21 ICR has been shown to activate the STAT3 pathway, thereby promoting Th17-like polarization of CAR-T cells *in vitro* and enhancing the toxicity of targeted HCC cells. IL-21 is the one that ultimately plays a direct role in promoting the anti-tumor function of CAR-T cells. A clinical trial of co-expressing IL-15 and IL-21 targeting GPC3 in multiple solid tumors (NCT04715191) is ongoing at Baylor College of Medicine. The objective of this study was to determine the maximum safe dose of CAR-T cells and to determine their survival time and side effects *in vivo*. At the same time, the efficacy was evaluated.

### IL-23

Gene-edited IL-23 CAR-T cells have been relatively infrequently studied, but have yielded significant results. Dawei Wang et al. (89) designed co-expressing IL-23 targeting prostate specific membrane antigen(PSMA) CAR-T cells and studied their antitumor functions. This study confirmed that *in vitro* proliferation and cytokine secretion of co-expressing IL-23 CAR-T cells were significantly higher than that of conventional CAR-T cells. Co-expressing IL-23 CAR-T cells also showed higher tumor clearance and faster weight recovery *in vivo*. Furthermore, it has been demonstrated that T cells upregulate IL-23α p19 subunit but not p40 subunit under TCR stimulation. Therefore, some researchers constructed CAR-T cells co-expressing the p40 subunit, and found that T cells obtained selective proliferative activity through the IL-23 signaling pathway. Compared with conventional CAR-T cells, P40-CAR-T cells showed superior antitumor activity (88). The therapeutic efficacy of p40-CAR-T cells in xenotransplantation of tumor-bearing mice was superior to that of conventional CAR-T cells.

## Structure Development and Optimization of Gene-Edited Interleukin CAR-T Cells

**Figure 1:**
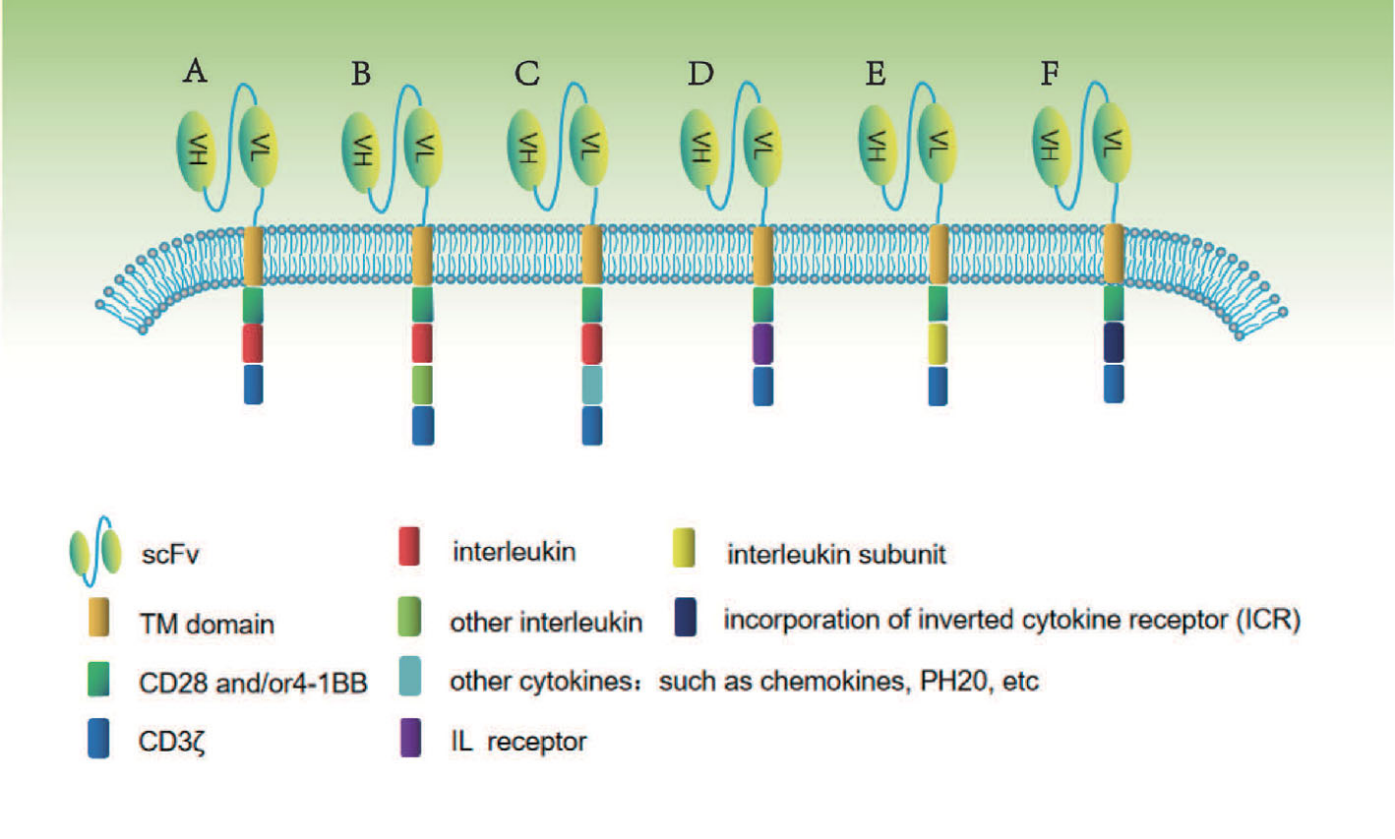
In this figure, different methods of constructing gene-edited interleukin-CAR T cells are shown. **(A)** Co-expression of a single interleukin. **(B)** Co-expression of two interleukins. **(C)** Co-expression of interleukin combined with other cytokines. **(D)** Co-expression of interleukin receptor. **(E)** Co-expression of interleukin subunit. **(F)** Co-expression of fusion interleukin ICR.

At present, the construction of gene-edited interleukin-CAR-T cell structure is diversified in the process of gradual optimization. The main construction methods for studying gene-edited interleukin-associated CAR-T include: co-expression of a single interleukin, two interleukin, interleukin combined with other cytokines, interleukin receptor, co-expression of interleukin subunit, and fusion ICR. The specific construction method is shown in **Figure 1**.

To enhance the tumor killing ability of CAR-T, researchers constructed CAR-T by gene-edited an interleukin that positively regulates T cell function, initially primarily for hematological tumors, and later for solid tumors. There are many relevant preclinical and clinical studies, as shown in **Tables 2** and **3**. For example, in 2018, Gray Kueberuwa et al. (79) constructed targeting CD19 IL-12-CAR-T cells in a preclinical study on the treatment of lymphoma, and the CAR-T cells expressing IL-12 in the trial not only killed CD19+ tumor cells directly, but also recruited other immune cells of the host for anti-tumor immune response. In 2020, Cong He et al. (75) constructed a CAR-T targeting NKG2D co-expressing IL-7, and in the treatment of prostate cancer, it was found that IL-7 production enhanced the expansion of CAR-T cells and inhibited their apoptosis. Later, researchers attempted to construct bileukin and interleukin combined with other cytokine CAR-T to enhance its tumor killing function. Andreas A. Hombach et al. (110) constructed co-expressing IL-7 and IL12 CAR-T cells, and the constructional production of IL-7 and IL-12 has been shown to enhance the expansion and persistence of CAR-T cells in preclinical studies of colorectal cancer. In 2018, Keishi Adachi team (74) constructed co-expressing IL-7 and CCL19 CAR-T cells, and demonstrated excellent tumor-killing activity in multiple solid tumors. Interestingly, researchers constructed both the co-expressing of IL-7 (IL-17-CAR) and the co-expressing of CCL19 (CCL19-CAR) T cells, and found *in vivo* that these two types of CAR-T cells were comparable to conventional CAR-T cells in killing tumors. This study demonstrates the limited ability of gene-edited individual interleukin CAR-T cells to enhance anti-tumor function. Furthermore, this suggests the importance of cytokine collaboration in enhancing CAR-T function. In 2020, Xingcheng Xiong and his team (76) constructed co-expressing IL-7 and PH20 CAR-T cells. Because the co-expressing PH20 can effectively degrade extracellular matrix, and enhance the tumor infiltration function of CAR-T cells. The study has demonstrated that co-expressing IL-7 and PH20 CAR-T cells can significantly improve their antitumor activity in multiple solid tumors. Therefore, the construction of multiple interleukin and interleukin combined with other cytokines gene-edited CAR-T cells is an important direction to conquer solid tumors in the future.

Side reaction should be considered while CAR-T cells improve immune function, after all, interleukin hypersaturation activation as cytokines is harmful to the body. Researchers constructed CAR-T cells that co-expressing interleukin receptors and applied the limited interleukin ligand in the tumor microenvironment to brake their functional release. Zhenhui Zhao et al. (77) constructed co-expressing IL-7 receptor(C7R) CAR-T cells, which shown good tumor killing effect *in vitro* in the preclinical experiment of treating triple-negative breast cancer. However, *in vivo*, C7R-CAR-T cells have not demonstrated any advantage over conventional CAR-T cells. Which may be influenced by the density of IL-7 ligand in tumor tissues. Xingcong Ma et al. (88) Constructed co-expressing IL-23 subunit (p40) CAR-T cells (p40-CAR-T) that in order to avoid the body damage caused by overactivation of cytokines. The results showed that p40-CAR-T cells had stronger antitumor activity compared to conventional CAR-T cells, and more importantly, showed fewer side effects compared to CAR-T cells co-expressing other interleukin *in vivo* trials. This study tells us that on the way to improve CAR-T function, we should not blindly increase the secretion of cytokines, but should achieve accurate co-expression and reduce meaningless harmful expression.

In the face of tumor inhibition microenvironment, most of the current studies are aimed at improving tumor killing functions by increasing the secretion of cytokines that positively regulate CAR-T function. However, this structural design ignores the value of immunosuppressive cytokines in the tumor immune microenvironment. Ann M Leen et al. (111) constructed CAR-T cells co-expressing the fusion ICR, and IL-4/IL-7 ICR (4/7 ICR) contained the IL-4 receptor ectodomain and the IL7 receptor endodomain. The study demonstrated that 4/7 ICR can be used to protect CAR-T cells from IL-4 inhibition. The 4/7 ICR accepts immunosuppressive IL-4 but converts its downstream signals into immune-stimulating IL-7 receptors. In contact with IL-4, CAR-T cells can maintain Th1 phenotype a strong antitumor activity *in vivo*. Then, Somala Mohammed et al. (90) generated CAR-T cells targeting prostate stem cell antigen (PSCA) 4/7 ICR-CAR-T cells, which demonstrated that 4/7 ICR-CAR-T cells grew normally in an IL-4-rich microenvironment, thereby enhancing their antitumor activity. Subsequently, Yi Wang et al. (85) reported a novel IL-4/IL-21 ICR (4/21 ICR) that improved the tumor killing efficacy of CAR-T cells through a mechanism different from that of the 4/7 ICR. This study demonstrated that 4/21 ICR activates the STAT3 pathway in response to IL-4 stimulation, promoting Th17-like polarization and tumor-targeted cytotoxicity of CAR-T cells *in vitro*. In addition, 4/21 ICR-CAR-T cells also showed strong antitumor activity against IL-4 positive tumors *in vivo*. Therefore, gene-edited ICR CAR-T cells are a promising clinical practice for the treatment of solid tumors.

## Potential Toxicity of Gene-Edited Interleukin CAR-T Cells

As mentioned above, gene-edited interleukin-CAR-T cell technology is optimized not only to enhance the function of CAR-T cells, but also to consider the cytotoxic effects of interleukin-over release. A phase 1 clinical trial of CD5-IL15/IL15 sushi CAR-T cells in refractory lymphoblastic lymphoma (NCT04594135) has been published (107). The patient was found to be well tolerated by infused CAR-T cells, causing only grade I CRS toxicity. Levels of ferritin and high-sensitivity C-reactive protein were briefly elevated. By detecting the cytokine level of patients in the first month, it was found that the expression of cytokines remained relatively stable. IL-15 levels also did not rise significantly after the infusion. CD5-IL15/IL15 sushi CAR-T cells secreted IL15/IL15 sushi complex in the body, which may lead to excessive IL-15 levels throughout the body. However, this was not observed in patients. This study demonstrates that gene-edited IL-15 CAR-T in the treatment of refractory lymphoblastic lymphoma causes mild CRS and is fully tolerated by the body. Besides, in the phase I clinical trial (NCT02587689) of MUC1-IL-12-CAR T cells constructed by Fengtao You for the treatment of seminal vesicle carcinoma, patients only started to experience mild headache, fever, muscle pain, nasal congestion and abdominal distention discomfort. From 6 to 12 days after the intratumoral injection, all discomfort disappeared and the body temperature returned to normal. Transient CRS was detected after intratumor injection, with a 10-fold increase in IL-6 and an approximately 60% increase in TNF-α (105). This study also confirmed that the side effects produced by MUC1-IL-12-CAR-T cells can be tolerated by the body. More clinical trials are needed to test the potential cytotoxicity of gene-edited interleukin-CAR-T cells before they can be widely used in the clinic.

## Discussion

Adoptive immunotherapy based on CAR-T cells has proved to be a promising strategy for the treatment of hematological malignant tumor. However, this clinical success has not been fully realized in solid tumors largely because of the hostile TME of solid tumors. Tumor immunosuppressive microenvironments limit the proliferation and persistence of CAR-T cells, and often impair the anti-tumor efficacy of CAR-T cells. Immunoregulatory cytokines, which are critical components of T cell activation, proliferation (10). Interleukin plays different roles in tumor immunity. They regulate the activation, proliferation and apoptosis of T cells, but also recruit peripheral immune cells to participate in tumor immunity. In the absence of these factors, even if the selected target is very good, CAR-T cells will not produce a complete and lasting killing effect on the tumor. Therefore, the above cytokines are used as cytokines for gene modification of CAR structures, and preclinical studies have also demonstrated that modified CAR-T cells can further enhance the efficacy of CAR-T cells by secreting cytokines.

In addition, the present study demonstrated that partial interleukin not only improves the function of CAR-T cells, but also engages the host peripheral immune cells to participate in the anti-tumor battle (79, 83, 100). This finding is critical because the current clinical use of CAR-T cell technology requires that host lymphatic clearance protocols provide adequate space for CAR-T. The current preclinical trial demonstrates that gene-edited interleukin-CAR-T cells can eliminate this step (79). This leads to the possibility that, on the one hand, the clinical treatment of the patient alleviates the pain of chemotherapy, and on the other hand, the anticancer activity of these immune cells can be utilized by genetically modifying IL secreted by CAR-T.

The CAR-T immunotherapy of genetically modified cytokines also faces the problem of dose limiting toxicity. When cytokines are produced in large quantities and corresponding receptors are reduced in the tumor microenvironment, peripheral tolerance is increased. Studies have demonstrated that genetically modified T cells lead to overexpression of the IL-7 receptor, thereby enhancing the antitumor activity of genetically modified IL-7 CAR-T and reducing the dose limiting toxicity (112). This may be one of the reasons why there are many studies on CAR-T co-expression of IL-7 at present. In response to this challenge, researchers developed CAR-T cells that genetically edited the interleukin-cell receptor and interleukin-subunit, which can effectively limit the over release of cytokines and prevent the development of CRS. However, the treatment of malignancies with gene-edited single interleukin CAR-T cells may also present problems of immune tolerance or cytokine inactivation. Therefore, the researchers began to gene-edited multiple cytokines to construct CAR-T cells, enabling the cytokines to enhance the synergistic action of CAR-T cells to kill tumor cells.

Gene-edited ICR CAR-T cells were developed to further enhance their antitumor activity while overcoming tumor immunosuppressor factors. The 4/7 ICR and 4/21 ICR CAR-T cell technologies rely on inhibitory regulatory cytokines to activate positive regulatory cytokines to perform immune regulatory functions. It can effectively reverse the inhibitory cytokine signal to the positive regulatory signal. Thus, CAR-T can better adapt to the tumor immunosuppressive microenvironment. However, the activation of ICR is limited by the expression of inhibitory factors in tumor tissues, and it is difficult to activate ICR once tumor tissues do not express targeted inhibitory cytokines. If combined with gene-edited interleukin and ICR to construct CAR-T cells, it may achieve the purpose of reversing the inhibitory signal and enhancing the positive signal. This may be an effective strategy for gene-edited interleukin-CAR T cells to conquer solid tumors. At present, there are a few studies in this area, and more preclinical studies are needed to verify its efficacy.

Currently, studies related to gene-edited interleukin CAR-T have achieved some results, but there is still a long way to go before it can be fully used in clinical trials. First, cytokines such as interleukin not only act on CAR-T cells, but also act on other immune cells, such as recruiting peripheral immune cells to participate in tumor immunity. However, it is difficult to achieve in immunocompromised mice, as part of the current pre-clinical trials are *in vivo* studies using immunocompromised mice. Secondly, the clinical treatment of gene-edited interleukin CAR-T has the possibility of CRS, because overstimulation of interleukin release, when tumor tissue receptor density cannot be satisfied, will inevitably increase the load of peripheral circulation. All the above need to be verified by further clinical studies. At present, most of the relevant clinical trials are in recruitment, and some of them have not been started yet. It is hoped that the relevant clinical research will achieve gratifying results.

## Conclusions

In summary, as immune regulatory factors, interleukin family members exert important functions in the activation and functional regulation of immune cells. In published preclinical and clinical studies, gene-edited interleukin CAR-T has been shown to enhance tumor killing in the treatment of malignancies with tolerable side effects. With the development of gene-edited technology and the development of researches on the interleukin family, gene-edited interleukin CAR-T technology in the treatment of malignant tumors will be able to achieve encouraging results.

## Author Contributions

All authors conceptualized and wrote the manuscript. LY conceived and modified the structure of this review. ZZ and ML additionally performed literature and data analysis. All authors contributed to the article and approved the submitted version.

## Funding

This work was supported by Special Research Project of Lanzhou University Serving the Economic and Social Development of Gansu Province (054000282), Lanzhou Talent Innovation and Entrepreneurship Project (2020-RC-38) and Fundamental Research Funds for the Central Universities (lzujbky-2020-kb14), Lanzhou Talent Innovation and Entrepreneurship Project (2020-28) and Major Science and Technology Special Project of Gansu Province (20ZD7FA003).

## Conflict of Interest

The authors declare that the research was conducted in the absence of any commercial or financial relationships that could be construed as a potential conflict of interest.

## Publisher’s Note

All claims expressed in this article are solely those of the authors and do not necessarily represent those of their affiliated organizations, or those of the publisher, the editors and the reviewers. Any product that may be evaluated in this article, or claim that may be made by its manufacturer, is not guaranteed or endorsed by the publisher.
